# Intracellular GSH Alterations and Its Relationship to Level of Resistance following Exposure to Cisplatin in Cancer Cells

**Published:** 2015

**Authors:** Bardia Jamali, Maryam Nakhjavani, Leila Hosseinzadeh, Salimeh Amidi, Nastaran Nikounezhad, Farshad H. Shirazi

**Affiliations:** a *Chemical Injuries Research Center, Baqiyatallah University of Medical Sciences, Tehran, Iran.*; b *Department of Toxicology and Pharmacology, School of Pharmacy, Shahid Beheshti University of Medical Sciences, Tehran, Iran.*; c *Department of Toxicology and Pharmacology, Faculty of Pharmacy, Kermanshah University of Medical Sciences, Kermanshah, Iran*.; d *Department of Medicinal Chemistry, School of Pharmacy, Shahid Beheshti University of Medical Sciences, Tehran, Iran. *; e *Pharmaceutical Sciences Research Center, Shahid Beheshti University of Medical Sciences,, Tehran, Iran.*

**Keywords:** A375, A2780s, A2780CP, HepG2, U373MGS, U373 MGCP, GSH, Cisplatin

## Abstract

One of the major complications in cancer chemotherapy with cisplatin as one of the important medicines in treatment regimens of different cancers is the development of resistance. One of the most described cellular defense mechanisms involved in resistance is glutathione (GSH), thus in this study, the effects of cisplatin on the total intracellular GSH level (GSHi) in some sensitive and resistant variants of human cell lines (hepatocarcinoma HepG2, skin A375, cisplatin sensitive glioblastoma U373MG and cisplatin resistant glioblastoma U373MGCP, cisplatin sensitive ovary A2780S and cisplatin resistant A2780CP cells) were studied. MTT assay was performed to measure cytotoxicity of cisplatin (33.3 µM for 1 hour). Following cisplatin exposure, GSHi (per million cells) was evaluated using a photometrical assay up to 90 minutes. Our results indicate that there are significant differences between GSHi content of A2780CP and U373MGCP cells compared to other cell lines. Moreover, IC_50_ of cisplatin in different cells seems to have a relation with mean of GSH level in 90 minutes (GSH (mean)_90_).

As a conclusion, it seems that resistance to cisplatin in different cell lines is more related with the diverse patterns of GSHi variations following cisplatin exposure than its original level, and/or its cellular increase or decrease. It is also suggested that GSH (mean)_90_ may be used as a factor for the prediction of cellular resistance to cisplatin.

## Introduction

Besides all the progress in cancer treatment (1), yet one of the biggest existing issues in chemotherapy is the development of resistance, which may result in treatment failure. Drug resistance occurs at the single cell level (2) and various mechanisms are proposed to describe the phenomenon (3). Thiol-mediated detoxificatioin of anticancer drugs is one of the important characterized drug-resistance mechanisms (4) and glutathione (GSH) is one of these important antioxidant defenses of the cell (5). The common characteristic of GSH substrates is the electrophilic structure, nitrogen mustards’ characteristics (4). For agents like cisplatin (6), which is known as a widely used anticancer drug against different types of cancer (7, 8), several studies indicate that its efficacy is limited by drug-induced resistance (9-11). In fact the cellular GSH content has always been associated with multidrug resistance (5) and in case of a drug like cisplatin that is believed to partially act the same as free radicals (12), resistance has always been related to elevated levels of intracellular GSH (10). Although many studies support this theory, others claim vise versa. Parsons and his coworkers believe that neither GSH level nor the enzymes that regulate it, are correlated with cellular resistance to alkylating agents (13) and Twentyman *et al.* indicate that increased GSH is not necessary for acquired cisplatin resistance (14). However, since GSH, as an important water phase antioxidant, plays an important role against radicals (15), formation of GSH-cisplatin conjugations is still one of the proposed mechanisms for detoxifying this medicine (16) and increased GSH levels seem to expand cells antioxidant defense and stabilize or raise cells threshold for susceptibility to toxic attack (15). Therefore, in this study, it was hypothesized that intracellular amount of GSH (GSHi), should be able to present a measurable scale of cellular resistance in different cell lines and that variations in cellular GSHi content and consumption after exposure to cisplatin may represent the degree of sensitivity and resistant to this drug in different cell lines. Consequently, in this study, the cytotoxicity of cisplatin in different cell lines, as well as the cellular GSHi levels after exposure to cisplatin is presented, to examine the accuracy of above hypothesis.

## Experimental

*Materials*

 Cisplatin powder was purchased from Sigma and diluted to the desired concentration with sterile 0.9 % sodium chloride. Cell culture media DMEM F12, antibiotic (streptomycin and penicillin), fetal bovine serum, HEPES buffer and trypsin were purchased from GibcoBRL. Standard GSH was purchased from Sigma. Tris buffer 1 N (pH = 8), TCA (Tricholoroacetic Acid) 10 % and DTNB (5,5'-dithiobis-(2-nitrobenzoic acid or Ellman's reagent) were purchased from Merck company. Solutions were prepared freshly for each set of experiment.

*Cell lines*

 A375 (human malignant melanoma cells, not responding to cisplatin based chemotherapy) and HepG2 (human hepatocarcinoma cells, a moderate responding cell line to cisplatin chemotherapy) were purchased from the Pasteur Institute of Iran. A2780S (human ovarian carcinoma-sensitive to cisplatin) and A2780CP (human ovarian carcinoma-resistant to cisplatin), U373MGS (human glioblastoma-sensitive to cisplatin) and U373MGCP (human glioblastoma-resistant to cisplatin) were obtained as a generous gift from Dr. Rakesh Goel, Ottawa Regional Cancer Center, Ottawa, Canada. 

*Methods*

*Cell culture*

 All cells were grown in DMEM/F12 Media supplied with 10% fetal bovine serum and 1% antibiotic. The cells were thawed and maintained in humidified 37 °C incubator with 5% CO_2 _for three passages before the start of experiments. 

*Measuring intracellular GSH content*

Cells were exposed to the 33.3 µM of cisplatin (as the human therapeutic serum concentration of this drug) for 15 minutes and GSH was then measured at 0, 15, 30, 45, 60 and 90 minutes, using of Ellmans method. Briefly, after exposure time, 10^6^ cells were centrifuged in 13500 rpm for 30 seconds, then the supernatant was removed and pellet was solved in 500 µL of distilled water. The cell lysate was treated with 10% TCA and incubated at room temperature for 30 minutes. The sample was again centrifuged in 13500 rpm for 30 seconds. 875 µL Tris buffer was added to 700 µL of supernatant before adding of DTNB (200 µL), and then the ultraviolet absorbance of samples was recorded at 412 nm. 

*Cytotoxicity MTT assay*

Cells were seeded in 96 well plates (6×10^6^ cell per well) overnight, and were then exposed to different concentration of cisplatin. After 1 hour, MTT was added to each well in darkness, and plates were incubated for 4 hours. Then the supernatant was discarded gently and 200 uL of dimethyl sulfoxide was added to each well. The plates were then shaken for 30 minutes and the optical density was recorded in Elisa plate reader (Biotech^®^) at 570 nm (17).

*Statistical analysis*

The results are expressed as mean ± standard error of the mean. Differences between means were evaluated by two way analysis of variance (ANOVA) by STATISTICA software (StatSoft, Inc., USA). The Newman-Keuls test was used for Post Hoc analysis. The p-value < 0.05 was considered as a significance level. IC_50_ was calculated by GraphPad Prism^®^ software (GraphPad Software, Inc., USA). 

## Results and Discussion

The role of GSH in the regulation of resistance to cisplatin has been explained by many researchers (18-20). Although it is stated that alteration in GSH level might not be the primary mechanism of resistance to cisplatin (21, 22), it is generally believed that high resistance to cisplatin is associated with increased synthesis of GSH (9, 23, 24). Yet, controversies in this area exist and all the cell lines do not have a similar pattern of resistance to this medicine. For example, Sandrine and coworkers showed that increase in GSHi did not influence resistance to cisplatin in Hela cell line (25). In the present study, the correlation between resistance to cisplatin and the pattern of intracellular GSH alterations was studied on some cisplatin resistant and sensitive cell lines. The clinical Minimum Residence Time (MRT) of cisplatin, after the injection of clinical doses is about 15 minutes and that is the exposure time we had selected for the observation of intracellular GSH variations. However, this exposure time is not enough to distinguish the level of resistance of this cell lines* in-vitro*. That is why the minimum more repeated exposure time in literatures of one hour has been selected to present statistically significant variations in these cell lines’ resistance to cisplatin. Ninety minutes is the length of observation for intracellular GSH that we could manage in the lab. Therefore, these timings are independent to each other. The selection of 15 minutes is to mimic the most relevant timing to the clinical exposure of tumor cells to cisplatin, 90 minutes observation was to follow the intracellular GSH alterations based on the general belief in clinical pharmacokinetics that the effect of a drug lasts for a minimum of 6 to 7 half lives (15 × 6 = 90). Sixty minutes exposure was the minimum time to observe the level of cellular resistance in the lab using MTT assay. To compare the intensity of resistance against cisplatin, MTT assay reveled IC_50_s of 0.9±0.1 µg/mL, 2±0.1 µg/mL, 2.3±0.1 µg/mL, 4.5±0.1 µg/mL, 2.8±0.1 µg/mL and 1.6±0.1 µg/mL for A2780S, A2780CP, U373MGS, U373MGCP, HepG2 and A375, respectively (Figure 2). These data confirm U373MGCP as the most resistant cell line in this study. 

**Figure 1:**
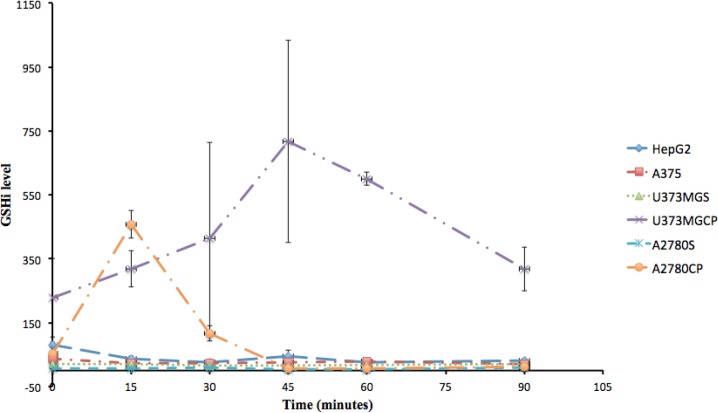
Kinetics of variations in intracellular GSH content (GSHi) of different cisplatin sesitive or resistant cell lines at 0, 15, 30, 45, 60 and 90 minutes after exposure to cisplatin.

Studying the intracellular content of GSH in each cell line showed that feature changes of GSHi content resistant cells (A2780CP and U373MGCP) are different from ovarian A2780, hepatic HepG2, skin A375 and gliblastoma U373MGS cells (P < 0.05) (Table 1). These differences were observed at 15 minutes for A2780CP and at 45 and 60 minutes for U373MGCP following the exposure to cisplatin (Figure 1). For ovarian cell lines, several studies have shown that there is a correlation between the degree of resistance to cisplatin and GSHi (26, 27). For example, it was previously reported that cisplatin resistant POE4 cell line has proportionally higher GSH levels than the sensitive POE1 cell line (28). In our study also, the GSHi level at base (time 0) for A2780CP was 10 times higher than A2780S and following exposure to cisplatin for 15 minutes, GSHi was further increased in the resistant type compared to the sensitive type. Abe *et al*. reported that in a 24-hour exposure to 1-300 µM of cisplatin, A2780CP shows 2.7 fold higher GSHi than A2780S (29). However, in our study, at the end of the 90 minutes, all cell lines, including A2780S and A2780CP, seemed to have the same amount of GSHi as their respective initial amount. Therefore it can be concluded that in addition to the initial content of GSHi, the resistance of cell lines are related to the pattern of increasing intracellular GSH in resistant type compared to sensitive cells.

**Table 1:** Intracellular GSH levels µM (mean ± standard error) in different cisplatin sensitive or resistant cell lines, at different intervals after exposure to cisplatin.

**Cell lines**	**Time (minutes)**					
	**0**	**15**	**30**	**45**	**60**	**90**
**HepG2**	80.73±8.04	37.44±7.24	26.09±5.34	44.26±20.65	25.29±1.32	30.63±6.6
**A375**	36.32±12.98	24.25±2.98	22.53±1.72	25.42±1.52	27.13±1.52	20.26±4.14
**U373MGS**	21.57±1.66	20.37±5.73	15.99±0.92	15.72±0.93	17.72±4.93	19.05±0.35
**U373MGCP**	228.1±122.98	318.14±56.28	414.28±299.51	716.78±315.82	600±20.41	318.14±68.93
**A2780S**	5.99±0.96	6.22±0.18	8.54±0.67	3.51±0.52	4.52±1.15	8.78±0.56
**A2780CP**	52.3±4.68	458.10±43.44	117.14±24.64	7.5±2.16	5.43±2.9	11.34±0.29

The initial (0 minutes) and the final (90 minutes) GSHi content of all understudied cell lines were statistically equal. Our results also indicate that not all resistant cell lines have high initial high level of GSHi. Although U373MGCP is a good example of a resistant cell line with high initial GSHi level compared to its sensitive strain, the initial GSHi level of A2780CP has no significant difference with its sensitive form, A2780S. Furthermore, the not-statistically significant difference of GSHi levels in sensitive cell lines during the time course of our study compared to fluctuations in GSHi content of resistant cell lines is another evidence conceding that not every cell line has a uniform, equal and alike pattern of GSHi level when it is exposed to cisplatin. Thus it can be concluded that the pattern of alterations in GSHi within time, can be a good indicator of resistance. Hence, what gives a better idea of the sensitivity or resistance ability of a cell might be the pattern of either GSHi fluctuations or its relatively steady state.

We were not able to determine a very specific correlation between cisplatin IC_50_ in all cell lines and their related GSHi levels. This diversity in cells resistances might be due to the complexity of factors involved in cisplatin resistance and the intrinsic biology of tumor type and cell line, as Chain and Waxman indicated (30). 

**Figure 2:**
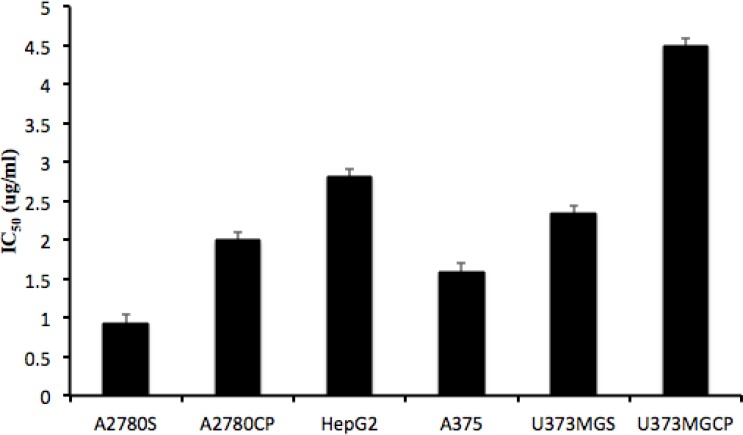
Comparing the IC50 (ug/mL) of cisplatin exposed to cisplatin sensitive or resistant cell line after 1 hour.

Furthermore, the fluctuations in GSHi of resistant cell lines, relatively steady pattern of GSHi content in sensitive cell lines, and comparing these patterns with the IC_50_ of cisplatin in these cell lines (Figure 2) confirm that different cellular pathways besides GSHi might be involved in resistance to cisplatin. 

Comparison of the mean GSHi in 90 minutes (GSH_(mean)90_) for each cell line with the related IC_50_ (µg/mL) of cisplatin, indicates that there is a correlation between resistance to cisplatin and GSH_(mean)90_ for each cell line (p-value=0.028), although no correlation exists between IC_50_ (µg/mL) and the time that intracellular GSH reaches the highest level in the first hour (min), the highest concentration of intracellular GSH, and GSH_(mean)60_ in our studied cell lines. 

## Conclusion

**Figure 3:**
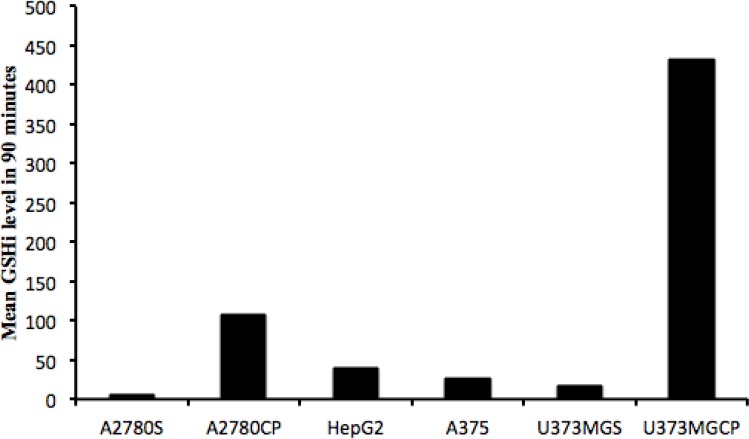
Comparing the mean intracellular GSH level in different cisplatin sensitive or resistant cell lines, up to 90 minutes after exposure to cisplatin.

With a comparison of Figures 2 and 3 it is obvious that within our test pairs of sensitive and resistant cell lines to cisplatin, the two pairs of U373MG and U373MGCP as well as the A2780S and A2780CP pairs were represented the best possible correlation between the GSHi and resistance to cisplatin, but not in a “one time point” manner. The best correlations for resistance and GSHi levels in these cell lines were observed for the mean of 90 minutes periods in which resistances (IC_50_) and GSH(mean)_90_ were very much representing each other with a p-value of 0.028.

As a conclusion, although it seems that considering the GSHi content in the tumor biopsy may be a great help to predict the resistance to cisplatin chemotherapy and might be a proximate guide for the selection of a more suitable chemotherapeutic choice, however, the mean GSHi content of the biopsy tissue cells exposed to cisplatin for a minimum of 90 minutes is much better predictor of resistant to cisplatin than the initial cellular content of GSHi. We are then proposing for the first time that the ability and speed of cells to respond to cisplatin with the increase in their GSHi levels is the most predictor of their resistance to cisplatin chemotherapy. Clinical investigations are needed to prove the accuracy of this finding on patients. 

## References

[1] Karol S, Pinedo Herbert M, Giaccone Giusseppe (1998). Drug resistance in Treatment of Cancer.

[2] Anderson B, Murray D, Steven T Rosen (2002). Clinically relevant resistance in cancer chemotherapy.

[3] Luqmani YA (2005). Mechanisms of drug resistance in cancer chemotherapy. Med. Princ. Pract.

[4] Tew KD (1994). Glutathione-associated enzymes in anticancer drug resistance. Cancer Res.

[5] Abdalla MY (2011). Glutathione as potential target for cancer therapy; more or less is good?. JJBS.

[6] Stewart JD, Bolt HM (2012). Cisplatin-induced nephrotoxicity. Arch. Toxicol.

[7] Shirazi FH, Wong PTT, Goel R (2003). Interaction of cisplatin with cellular macromolecules: a fourier transform infrared spectroscopy study. Iran. J. Pharm. Res.

[8] Fahimi F, Khodadad K, Amini S, Naghibi A, Salamzadeh J, Haghgoo R, Baniasadi S (2011). Evaluating the effect of zingiber officinalis on nausea and vomiting in patients receiving cisplatin based regimens. Iran. J. Pharm. Res.

[9] Godwin AK, Meister A, O'Dwyer PJ, Huang CS, Hamilton TC, Anderson ME (1992). High resistance to cisplatin in human ovarian cancer cell lines is associated with marked increase of glutathione synthesis. Proc. Natl. Acad. Sci.

[10] Kasherman Y, Sturup S, Gibson D (2009). Is glutathione the major cellular target of cisplatin? A study of the interactions of cispatin with cancer cell extracts. J. Med. Chem.

[11] Galluzzi L, Senovilla L, Vitale I, Michels J, Martins I, Kepp O, Castedo M, Kroemer G (2012). Molecular mechanisms of cisplatin resistance. Oncogene.

[12] Matsushima H, Yonemura K, Ohishi K, Hishida A (1998). The role of oxygen free radicals in cisplatin-induced acute renal failure in rats. J. Lab. Clin. Med.

[13] Parsons PG, Lean J, Kable EP, Favier D, Khoo SK, Hurst T, Holmes RS, Bellet AJ (1990). Relationships between resistance to cross-linking agents and glutathione metabolism, aldehyde dehydrogenase isozymes and adenovirous replication in human tumour cell lines. Biochem. Pharmacol.

[14] Twentyman PR, Wright KA, Rhodes T (1991). Radiation response of human lung cancer cells with inherent and acquired resistance to cisplatin. Int. J. Radial. Oncol.

[15] Kidd PM (1997). Glutathione: systemic protectant against oxidative and free radical damage. Altern. Med. Rev.

[16] Komiya S, Gebhardt MC, Mangham DC, Inoue A (1998). Role of glutathione in cisplatin resistance in osteosarcorna cell lines. J. Orthop. Res.

[17] Faedmaleki F, Shirazi FH, Salarian A, Ahmadi Ashtiani H, Rastegar H (2014). Toxicity effect of silver nanoparticles on mice liver primary cell culture and HepG2 cell line. Iran. J. Pharm. Res.

[18] Chen HHW, Kuo MT (2010). Role of glutathione in the regulation of cisplatin resistance in cancer chemotherapy. Metal-Based Drugs.

[19] Siddik ZH (2003). Cisplatin: mode of cytotoxic action and molecular basis of resistance. Oncogene.

[20] Kuo MT (2009). Redox regulation of multidrug resistance in cancer chemotherapy: molecular mechanisms and therapeutic opportunities. Antioxid. Redox. Signal.

[21] Pratesi G, Dal Bo L, Paolicchi A, Tonarelli P, Tongiani R and Zunino F (1995). The role of the glutathione-dependent system in tumor sensitivity to cisplatin: A study of human tumor xenografts. Ann. Oncol.

[22] Zendehdel R, Masoudi-Nejad A, Mohammadzadeh J, Shirazi FH (2012). Cisplatin resistant patterns in ovarian cell line using ftir and principle component analysis. Iran. J. Pharm. Res.

[23] Micetich K, Zwelling LA, Kohn KW (1983). Quenching of DNA: Platinum(II) monoadducts as a possible mechanism of resistance to cis-Diamminedichloroplatinum(II) in L1210 Cells. Cancer Res.

[24] Hromas RA, Andrews PA, Murphy MP and Burns CP (1987). Glutatione depletion reverses cisplatin resistance in murine L1210 leukemia cells. Cancer Lett.

[25] Daubeuf S, Leroy P, Paolicchi A, Pompella A, Wellman M, Galteau MM and Visvikis A (2002). Enhanced resistance of HeLa cells to cisplatin by overexpression of gama-glutamyltransferase. Biochem. Pharmacol.

[26] Meurette O, Lefeuvre-Orfila L, Rebillard A, Lagadic-Gossmann D and Dimanche-Boitrel MT (2005). Role of intracellular glutathione in cell sensitivity to the apoptosis induced by tumor necrosis factor alpha-related apoptosis-inducing ligand/ anticancer drug combinations. Clin. Cancer Res.

[27] Wang W, Sun YP, Huang XZ, He M, Chen YY, Shi GY, Li H, Yi J and Wang J (2010). Emodin enhances sensitivity of gallbladder cancer cells to platinum drugs via glutathion depletion and MRP1 downregulation. Biochem. Pharmacol.

[28] Lewis AD Hayes JD, Wolf CR (1998). Glutathian and glutathione dependent enzymes in ovarian adenocarcinoma cell lines derived from patients before and after the onset of drug resistance. Carcinogenesis.

[29] Abe T, Gotoh S, Higashi K (1999). Attenuation by glutathione of hsp72 gene expression Induced by cadmium in cisplatin-resistant human ovarian cancer cells. Biochem. Pharmacol.

[30] Chen G, Waxman DJ (1994). Role of cellular glutatione and glutathione S-transferase in the expression of alkylating agent cytotoxicity in human breast cancer cells. Biochem. Pharmacol.

